# Evaluating the reliability of the Attitudes to Randomized Trial Questionnaire (ARTQ) in a predominantly African American sample

**DOI:** 10.1186/s40064-015-1208-z

**Published:** 2015-08-12

**Authors:** Marvella E Ford, Wei Wei, Leslie A Moore, Dana R Burshell, Kimberly Cannady, Franshawn Mack, Nnadozie Ezerioha, Kelley Ercole, Elizabeth Garrett-Mayer

**Affiliations:** Cancer Disparities Program, Hollings Cancer Center, Medical University of South Carolina, Charleston, SC USA; College of Medicine, Medical University of South Carolina, Charleston, SC USA; College of Nursing, Medical University of South Carolina, Charleston, SC USA; South Carolina State University, Orangeburg, SC USA; Department of Public Health Sciences, Medical University of South Carolina, Charleston, SC USA; College of Charleston, Charleston, SC USA

**Keywords:** African Americans, Cancer, Clinical trials, Perceptions, Psychometric testing

## Abstract

**Purpose:**

To evaluate the reliability of the Attitudes to Randomized Trial Questionnaire (ARTQ) in measuring perceptions of cancer clinical trials in a predominantly African American (AA) sample in South Carolina (SC).

**Methods:**

Principal Component Analysis (PCA) and Cronbach’s alpha estimates were used to assess the reliability of the ARTQ in a convenience sample of 315 participants (81.4 % AA) who were recruited from 2008 to 2013, and who live in eleven different counties in South Carolina with high rates of racial disparities in cancer mortality rates.

**Results:**

Slightly more than half of the 315 participants had at least a college education (77.9 %), 84.8 % were female, and 53.1 % had an annual income of $40,000 or more. In this study, PCA confirmed that the ARTQ is unidimensional. Cronbach’s alpha for the ARTQ was 0.86.

**Conclusion:**

The ARTQ displayed strong evidence of high statistical reliability. This analysis has great implications for future research because it represents the first test of reliability of the ARTQ in a predominantly African American sample and lays the groundwork for use of the ARTQ in future studies in diverse populations.

## Background

Cancer is the second leading cause of death in the United States where it accounts for more than one million deaths per year. For the majority of cancer types, African Americans have the highest cancer mortality rate of any other racial or ethnic group in the United States (Seigel et al. 2014). For example, in breast cancer, African American women have a 28 % higher death rate as compared to European Americans (EA) despite a 13 % lower rate of developing breast cancer (Harris et al. 2003). In South Carolina (SC), rates of cancer related deaths are among the highest in the nation, particularly among African Americans (Seigel et al. 2014). A possible contributor toward these disparities could be negative perceptions of cancer and cancer clinical trials (Fleissig et al. 2001; Ramirez et al. 2005; Seigel et al. 2014; Ford et al. 2012; Langford et al. 2010). Testing the reliability of the ARTQ in African American populations will indicate if the ARTQ is an effective tool for insight into this particular population’s ideas about clinical trials and willingness to participate.

### Conceptual framework

Previous studies show that members of African American (AA) communities may require additional knowledge about cancer screening, prevention, early detection, and treatment. Low levels of knowledge are associated with low self-efficacy and low rates of participation in prostate, breast and cervical cancer screening (Barber et al. 1998; Glick et al. 2012). For example, many AA men report that clinicians do not communicate effectively with them about prostate cancer screening (Sellers and Ross 2003). Lack of knowledge precludes patients’ feelings of self-efficacy to actively engage in shared decision making about screening with their clinicians. Therefore, as cancer knowledge increases, participants’ confidence in their ability to effectively communicate with their clinicians about cancer would be expected to increase commensurately (Sellers and Ross 2003).

The need to expand the knowledge base of cancer clinical trials among diverse community members is underscored by Ford et al. (2012) who reviewed sixty-five studies focusing on recruitment of racially and ethnically diverse participants to cancer clinical trials. Lack of education regarding cancer clinical trials was the most frequently reported barrier to participation (Ford et al. 2012). Similarly, Langford et al. (2010) reports that lack of knowledge about clinical trials, and subsequent negative perceptions of them, are formidable barriers to the participation of diverse populations in trials.

**Fig. 1:**
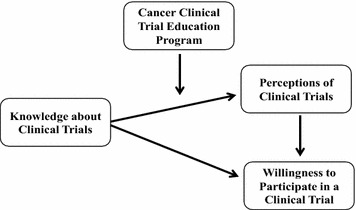
Conceptual framework of perceptions of clinical trials.

Thus, as shown in Fig. 1, lack of knowledge about trials can lead to negative perceptions of them, which in turn has a negative impact on trial participation. Unfortunately, negative perceptions of cancer clinical trials based on lack of knowledge can negatively impact trial recruitment in the very populations that could most benefit from the scientific knowledge gained through their participation (Fisher and Kalbaugh 2001). Fallowfield et al. (1998) argue that recruitment difficulties often arise from potential participants’ lack of understanding of terms such as “randomization.” Misperceptions in the randomization process (i.e., for participants with cancer, the minimum level of care received is the best available current treatment rather than placebo) can also lead to suspicion on the part of potential participants about the ethical nature of the research (Fleissig et al. 2001; Ford et al. 2008).

### Rationale for testing the reliability of the ARTQ in a predominantly African American sample

While greater participation in cancer clinical trials among AAs could help to reduce this disparity, negative perceptions of trials may play a role in negatively impacting trial participation in this population. The objective of this study is to evaluate the reliability of the Attitudes to Randomized Trial Questionnaire (ARTQ) in assessing perceptions of cancer clinical trials in predominantly AA populations in South Carolina (SC). The ARTQ was developed in Europe and has not yet been tested for use in an AA sample (Fallowfield et al. 1998).

Before clinical trial perceptions among AAs can be improved, investigators must first understand the attitudes of the AA population towards these trials. However, in order for health disparities research to be conducted in a meaningful manner, it is important to determine first whether measures developed among non-minority populations perform in the same way when applied to minority populations.

The results of the seven-question survey called the Attitudes to Randomized Trial Questionnaire (ARTQ) developed and tested in Europe, suggest that most patients have positive perceptions of clinical trials and were willing to consider trial participation (Fallowfield et al. 1998). Analysis demonstrated that this instrument was reliable in a European population in evaluating the perceptions of study participants towards clinical trials (Fallowfield et al. 1998; Jenkins et al. 2010). This tool may be useful in assessing perceptions of clinical trials in the AA population and has been used in previous studies (Ford et al. 2012). However, the reliability of this instrument has never before been tested in a US or an AA sample and its applicability in this population is unclear. Kidder noted that instruments tested and developed in one type of population may show high reported reliability in that population but low reliability when applied to another population (Kidder et al. 1986; Kimberlin and Winterstein 2008). Therefore, before the ARTQ is widely used in AA populations in future studies, it is important to confirm the reliability of this instrument in an AA population, and compare it with reliability results of the instrument in EA populations (Kimberlin and Winterstein 2008).

The factorial structures of health measures may vary across racial Groups (Seigel et al. 2014). It has been noted that self-reports of health by older AA and Caucasian adults do not merely reflect clinical status or objective health but also the influences of cultural and social factors (Ford et al. 2004). For example, Gibson used latent variable confirmatory factor analysis to examine racial differences in the structure and measurement of self-reports of health widely used in studies involving older adults (Gibson 1991).

As Gibson (1991) concluded, additional factors unique to each racial group that influence subjective interpretation of health state could be modeled; supporting that simply using the same measurement instrument for older AAs and Caucasians and comparing the results may reflect not only a true racial difference but also differences in the reliability and validity of the measurement instrument or its underlying construct(s) for the two groups (Gibson 1991; Kimberlin and Winterstein 2008). Therefore, the objective of this study was to evaluate the reliability of the ARTQ in a sample of AAs who were recruited at multiple Train the Trainer intervention sites in South Carolina.

## Methods

### Study sample and rationale for site selection

The clinical trial education program was part of a larger 4-h evidence-based cancer education program in which a 3-h component focused on general cancer knowledge, a 30-min component focused specifically on prostate cancer knowledge, and a 30-min component focused on cancer clinical trials knowledge. A pretest/posttest design was also used to determine the effectiveness of the program. Only pre-test data were used in this analysis.

**Fig. 2:**
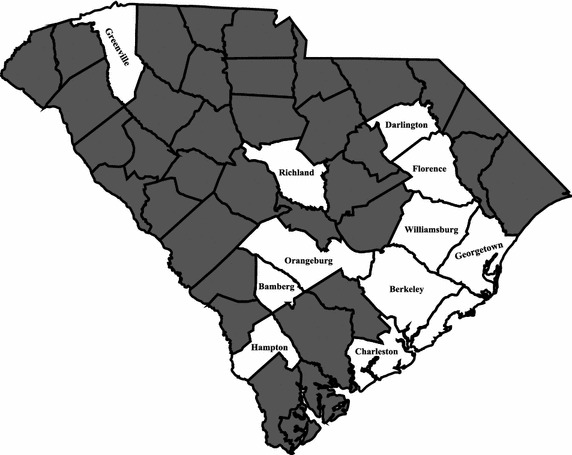
Program sites in South Carolina.

The cancer clinical trial education program was conducted at ten sites in eleven different counties representing several different geographic regions of the state (Fig. 2). These eleven counties (and sites) include Berkeley (Varnertown Indian Community), Georgetown (Georgetown), Charleston (Charleston and Johns Island), Greenville (Greenville), Orangeburg (both Orangeburg sites), Richland (Columbia), Bamberg (Denmark), Florence (Florence), Darlington (Darlington), Hampton (Yemassee), and Williamsburg (Kingstree). The study included a convenience sample of participants in communities with high racial disparities in cancer mortality rates. The counties where the intervention was conducted seem to be clustered in certain areas because of the word-of-mouth response to the program. Because it was conducted in certain counties, representatives of neighboring counties have asked for the program to be conducted in their counties as well.

As shown in Table 1, which describes 2007 cancer mortality rates for AAs versus EAs, the rates are significantly higher for AAs. In South Carolina, the cancer mortality rate for AAs is 223.1, compared with 183.2 for EAs. Table 2 describes the demographic characteristics of the counties where the cancer education program was conducted. As may be seen, the majority of the counties have median household incomes and per capita incomes below that of the United States or even of South Carolina as a whole. As will be described (see section entitled “community engagement activities conducted in the study”), a variety of community engagement strategies were employed to recruit participants in these identified communities.

**Table 1:** Age-adjusted cancer mortality rates for the counties where the program was conducted

Race	County name											
	South Carolina (Ref)	Bamberg	Charleston	Darlington	Dorchester	Florence	Georgetown	Greenville	Hampton	Orangeburg	Richland	Williamsburg
European American	167.9	n/a^a^	158.3	217.1	171.1	156.4	187.8	165.1	209.5	155.9	156.9	182.9
African American	210.9	275.3	239.3	227.1	182.3	229.6	228.2	180.8	259.6	250.8	253.5	208.7

Source: Division of Biostatistics and Health GIS, PHSIS, SC DHEC; 2009 Cancer Mortality data using age adjusted rates from the 2000 US Standard Population.

^a^n/a due to the low number of cancer deaths in the EA population in Bamberg.

**Table 2:** Demographic characteristics of the counties where the program was conducted

County name	Racial composition										
	Size	European American	African American	American Indian/Alaskan Native	Asian	Native Hawaiian/Pacific Islander	≥2 races	Hispanic/latino	Median household income ($)	Per capita income ($)	Population below the poverty level (%)
US (Ref.)	316.5 million	77.7	13.2	1.2	5.3	0.2	2.4	17.1	53,046	28,155	15.4
South Carolina (Ref.)	4.8 million	68.3	27.9	0.5	1.5	0.1	1.7	5.3	44,779	23,943	18.1
Bamberg (Denmark)	15,430	36.8	61.4	0.4	0.5	0.1	0.9	1.8	31,483	18,902	27.6
Berkeley	194,020	69.1	25.0	0.7	2.5	0.1	2.6	6.1	52,427	24,165	13.8
Charleston	372,803	67.4	29.0	0.4	1.6	0.1	1.5	5.2	50,792	30,158	18.2
Darlington	67,935	56.7	41.3	0.4	0.5	^a^	1.1	1.9	36,323	20,105	24.0
Florence	138,326	54.9	42.0	0.4	1.4	^a^	1.2	2.4	41.910	22,432	19.8
Georgetown	60,440	65.4	32.8	0.3	0.6	^a^	0.9	3.0	40,131	24,437	21.2
Greenville	474,266	77.1	18.5	0.5	2.2	0.1	1.7	8.7	49,022	26,643	15.8
Hampton	20,408	44.1	53.7	0.4	0.6	^a^	1.1	3.8	34,233	19,332	25.2
Orangeburg	90,942	34.9	62.3	0.6	0.9	^a^	1.3	2.1	34,110	17,687	23.7
Richland	399,256	48.1	46.8	0.4	2.6	0.1	2.0	5.0	48,359	25,763	17.2
Williamsburg	33,067	32.9	65.5	0.4	0.4	^a^	0.8	2.2	25,849	14,845	30.8

Available from http://quickfacts.census.gov/qfd/states/45000.html.

^a^Value greater than zero but less than half unit of measure shown.

In South Carolina, racial differences are seen in cancer related behaviors. These differences could contribute to the disparities in cancer mortality rates that are shown in Table 1. For example, in South Carolina in 2009, 20.6 % of EAs smoked, versus 21.4 % of AAs; 64.2 % of EAs were overweight/obese, compared with 75.7 % of AAs; among the non-elderly population in the state, only 47 % of AAs had employer-sponsored health insurance coverage, compared with 64 % of EAs; and among the non-elderly population in the state, 46 % of EAs versus 49 % of AAs had Medicaid coverage (Neighbors et al. 2003). These data show that AAs are more likely than EAs to have higher rates of risk factors for cancer and at the same time, lower rates of access to care (Prevention 2011; Reid et al. 2010).

- Berkeley (Varnertown Indian Community)
- Georgetown (3 in Georgetown)
- Charleston (2 in Charleston and 1 in Johns Island)
- Greenville (Greenville)
- Orangeburg (2 in Orangeburg and 1 in Santee)
- Richland (Columbia)
- Bamberg (Denmark)
- Florence (Florence)
- Darlington (Darlington)
- Hampton (Yemassee)
- Williamsburg (Kingstree)

### Measures

The ARTQ, a seven-item instrument developed by Fallowfield et al. was used to assess perceptions of cancer clinical trials (Fallowfield et al. 1998). Table 3 lists the instrument questions. Question 1 inquires if patients/survey participants should be asked to take part in medical research. Questions 2 and 3 inquire if the survey participant would consider enrolling in clinical research comparing treatments. Question 3 explores the participant’s response if the study were a treatment chosen at random. In Questions 4–6, participants are asked to read three statements providing more information about research studies: (1) both treatments were completely suitable for the participant (Question 4); (2) a study participant could leave the study if the treatment did not suit him or her (Question 5); and (3) the doctor would explain both treatments prior to the participant being randomized (Question 6). Participants are then asked if knowing this additional information would encourage them to take part in a clinical trial. Question 7 asks whether knowing the extra information from the three previous statements would affect their willingness to participate in a clinical trial (Fallowfield et al. 1998). The ARTQ questions were read to the group of participants, and individuals recorded their responses, “Yes,” “No,” or “Do Not Know,” on paper copies which were subsequently de-identified. Additional survey items assessed general demographic characteristics of participants, including Hispanic ethnicity, race, highest level of education completed, marital status, household income, age, and gender.

**Table 3:** Seven-item Attitudes to Randomized Trials Questionnaire (ARTQ)

Previous validation showed that the ARTQ scores predicted trial participation [12]
The ARTQ is interviewer administered. Responses include Yes, No, Do Not Know (DK)
Perceptions of clinical trials
1. Do you think that patients should be asked to take part in medical research?
2. Suppose that you were asked to take part in a research study comparing two treatments, both of which were suitable for your illness. Would you be prepared to take part in a study comparing different treatments?
3. Usually the only scientific way to compare one treatment with another is for the choice between the two to be made randomly, rather like tossing a coin. Would you be prepared to take part in a study where treatment was chosen at random?
4. If you answered “No” or “DK” to Question 3, we would now like to ask you a bit more about this. In a randomized study a choice would be made between two treatments, either of which would be suitable for you. Your doctor and experts in the field do not know for sure if one treatment is better than the other, or if they are both the same. That’s why they want to do the study. Would knowing that encourage you to take part?
5. In a random choice study, if the treatment you were receiving did not suit you for any reason you could leave the study. Your doctor would then give you whatever treatment might be appropriate for you. Would that encourage you to take part?
6. Before you agreed to enter a random choice study the doctor would tell you all about the two treatments being compared, before you were allocated to one or the other. Would that encourage you to take part?
Intention/willingness to participate in a clinical trial
7. If you knew all the following things were taken in account, would you change your mind and agree to take part in the study? Both treatments were completely suitable. You could leave the study if the treatment did not suit you. There is plenty of information before the random choice was made

### Statistical analysis

The survey data were double-entered into SPSS 21 and were compared for verification of data entry. Data were exported to a comma-separated format and analyzed using the R statistical package (R version 2.3.0). Responses were coded as “Yes,” “No,” and “Don’t Know.”

Two approaches were considered for handling the three-level response variable. The first approach treated response as a binary variable by coding “don’t know” as missing, whereas the second approach took response as a categorical variable with three levels. Our primary inferences are based on treating “Don’t Know” as “missing.” The latter approach was used as a sensitivity analysis to evaluate how sensitive our inferences were to the different approaches for coding responses. Using the recoded data, tetrachoric correlations were calculated, which convert a measure of association (the odds ratio) between two binary measures to a correlation (−1 to 1) scale.

Principal components analysis was then applied to the correlation matrix. The eigenvalues from the principal components analysis were evaluated to determine the dimensionality of the scale. After concluding that unidimensionality of the items was appropriate, Cronbach’s alpha was used to measure the internal consistency reliability of the items. Several approaches were considered for handling the three-level variable, including combining “Don’t Know” with “No”, recoding “Don’t Know” as missing, and using a polychoric correlation. Our primary inferences are based on replacing “Don’t Know” with “No.”

## Results

Since our analysis only focused on the binary Yes/No responses to the ARTQ items, the “Don’t Know” responses were coded as missing in the calculation of the Cronbach’s alpha.

Table 4 shows the demographic characteristics of all participants (n = 315). Among those who reported data for each category the majority were AA (81.4 %) and female (84.8 %). Slightly more than 60 % of participants had at least a college degree. About half were either widowed, divorced, separated, or never married (53.3 %), and almost half had an annual household income of more than $40,000 (53.4 %).

**Table 4:** Summary of demographic characteristics of participants at pretest (N = 315)

Variable	n (%)
Race^a^ (n = 296)	
African American	241 (81.4)
Native American/Alaskan native	15 (5.1)
European American	40 (13.5)
Gender^a^ (n = 211)	
Male	32 (15.2)
Female	179 (84.8)
Hispanic^a^ (n = 298)	
Yes	6 (2.0)
No	292 (98.0)
Age^a^ (n = 297)	
<50	137 (46.1)
51–64	107 (36.0)
65–75	45 (15.1)
76+	8 (2.7)
Education level^a^ (n = 298)	
<8 years	4 (1.3)
8–11	11 (3.7)
12 years or HS completion	31 (10.4)
Post-HS other than college	20 (6.7)
Some college	51 (17.1)
College graduate	100 (33.6)
Postgraduate	81 (27.2)
Marital status^a^ (n = 297)	
Married or living as married	139 (46.8)
Widowed	24 (8.1)
Divorced	48 (16.2)
Separated	9 (3.0)
Never married	77 (26.0)
Income^a^ (n = 286)	
$0–$19,999	66 (23.1)
$20,000–$39,999	67 (23.4)
$40,000–$59,999	61 (21.3)
$60,000–$79,999	43 (15.0)
$80,000+	49 (17.1)

*HS* high school.

^a^Missing data.

Table 5 shows a comparison of the study demographics to South Carolina’s demographics using the 2010 South Carolina Census data (Bureau 2010, 2015).

**Table 5:** Comparison of study participant demographics (N = 315) to South Carolina (SC) demographics (N = 4,625,364)

Variable	Study %	SC %
Race^a^ (n = 296)		
African American	81.4	28.0
Native American/Alaskan native	5.1	0.5
European American	13.5	68.4
Gender^a^ (n = 211)		
Male	15.2	48.6
Female	84.8	51.4
Hispanic^a^ (n = 298)		
Yes	2.0	5.3
Age^a^ (n = 297)		
≥65	17.8	14.7
Education level^a^ (n = 298)		
College graduate	60.8	24.2
Marital status^a^ (n = 297)		
Married or living as married	46.8	59.0
Income^∆^ (n = 286) see note below		
The median income of the study participants fell between $40,000 and $59,000. The median income in SC is $44,587		

South Carolina 2010 census data.

*HS* high school.

^a^Missing data.

**Fig. 3:**
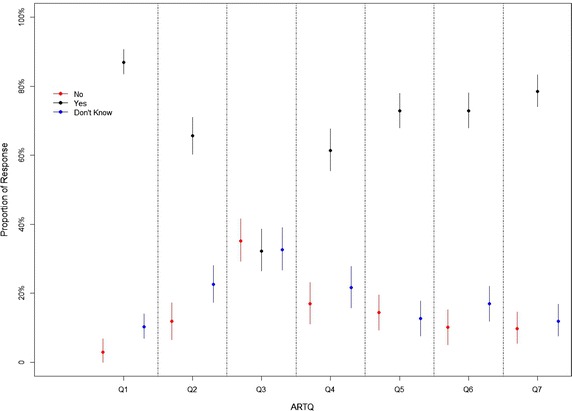
Proportion of participants who responded Yes/No/Don’t Know for the seven items in the attitudes to randomized trials questionnaire.

Figure 3, made with the R 3.1.2. Software shows the proportion of participants who responded Yes/No/Don’t Know for the seven items in the ARTQ. The proportion of Yes is higher in all questions (≥66 %) compared to No/Don’t know with the exception of question 3 (Y = 32 %, N = 35 %, DK = 33 %).

### Psychometric properties of the ARTQ

There was strong evidence of unidimensionality: the first eigenvalue in PCA was 4.5, the second was 0.76. This can be interpreted to mean that 64 % (4.5/7) of the variance in the seven items is explained by a combined composite score. As a result, it is appropriate to assume the items are measuring the same construct and to evaluate the internal consistency reliability of the items. This was done by using Cronbach’s alpha, which was 0.86, indicating high reliability. Sensitivity analysis was implemented and results were similar, suggesting the handling of “don’t know” responses had little effect on the reliability.

### Limitations

It is unclear whether similar responses would have been seen in a sample that had a greater proportion of men. However, in our previous study focusing on recruitment of AA men to a cancer clinical trial, female spouses or partners were found to serve as “gatekeepers” in terms of access to the male study participants. The prior study also showed that women transmitted health information to the men in their lives (Ford et al. 2001, 2008; Griffith et al. 2012). Therefore, in the present study, although efforts were made to include men by publicizing the cancer education sessions in each area with male-dominated organizations such as fraternities, masonic orders, and ministerial alliances, the investigators felt confident that the women who participated in the sessions would share the information with their husbands, sons, nephews, and others.

## Discussion

The results of this study show that the ARTQ is a reliable instrument in the AA sample and could be used to assess perceptions of cancer clinical trials among AAs in future studies. The ARTQ showed high percentages of Yes responses to all items with the exception of Question 3. This shows that most participants are generally are willing participants in clinical trials if they are approached.

An important finding emerged related to the dimensionality of the ARTQ. Previous studies with predominantly EA samples showed that the ARTQ exhibited two factors: perceptions of clinical trials and willingness to participate in a clinical trial. However, in the present study, the results showed that the ARTQ exhibited unidimensionality, with only one factor—perceptions of clinical trials. Our previously published data showed that for each of the seven items on the ARTQ, responses changed in a more positive direction from pre-intervention to post-intervention (p < 0.01) (M. Ford et al. 2012). Thus, the intervention resulted in more favorable perceptions and greater willingness to participate in a trial if offered. However, the psychometric analyses of the present study showed that willingness to participate was not a separate factor on the scale in this predominantly AA sample.

No other studies reported in the literature have evaluated the ARTQ in assessing perceptions of trials among AAs, as the majority of studies using the ARTQ have been conducted in Europe. As such, the present study makes an important contribution to the research literature. This contribution is the development of culturally equivalent measures and signifies a step forward in the accurate assessment of health, health determinants and outcomes in the context of multicultural research, thus potentially contributing to the alleviation of health disparities.

While prior published results indicated the ARTQ showed high reliability in EA populations, its reliability needed to be tested in an AA population. Substantial differences related to health outcomes have been observed across different ethnic/racial groups (Ramirez et al. 2005). However, it is uncertain whether these observed differences reflect true differences, or whether they merely reflect cultural bias in the measures (Liang et al. 1987; Neighbors et al. 2003; Ramirez et al. 2005). The presumption of social or cultural homogeneity exacerbates inaccurate cultural stereotypes, can lead to misleading conclusions in comparing prevalence of disorders, and can hinder the delivery of quality health care to different racial and ethnic groups.

In the context of cross-cultural comparison, an important factor is consideration of the population of origin for instrument development, and whether the instrument has been tested for use in other populations. Instruments that are not validated with respect to a particular racial or ethnic group are likely to carry different psychometric properties than is the one originally developed. For example, Fillenbaum et al. examined seven cognitive screening or neuropsychological tests in relation to clinical diagnosis (Fillenbaum et al. 1990). The authors reported that most measures, when adjusted for race and education, had lower specificities for AAs than for whites (Fillenbaum et al. 1990).

Patients may decline entry into randomized clinical trials because of uncertainty about personal benefit (Llewellyn-Thomas et al. 1991), concerns as to whether or not the best available treatment would be given (although it has been shown that trial participation leads to better outcomes and overestimation of the likely therapeutic benefits of standard therapy) (Sheldon et al. 1993). Poor understanding about the value of clinical trials specifically and experiments in medicine in general, produces suspicion and confusion among the general population. This may help to explain why the proportions of Yes/No/Don’t Know responses to ARTQ Item 3 were so close together. It is important to note the ethnicity of the study’s participants. The percentages of AAs and Native Americans/Alaskan Natives in the study are substantially higher than the state population percentage. In South Carolina the state percentage of AAs is 28 %, whereas in study sample it was 81.4 %. The state percentage of Native Americans/Alaskan Natives is 0.5 % and in the study sample it was 5.1 %. These data reflect the participants’ willingness to participate in the intervention at rates higher than their representation on the state level.

It is possible that the outcomes of the Train the Trainer program are attributable to the relatively high level of education of the participants. According to the 2010 U.S. Census Bureau estimates, only 24.2 % of the South Carolina population over the age of 25 has completed their bachelor’s degree. In the present study, 33.6 % of the participants had obtained a college diploma and an additional 27.2 % had completed postgraduate education. Thus, the participants in this study were more highly educated than the general population of South Carolina. However, the incorporation of the “Train the Trainer” approach means that the participants are now equipped to administer the intervention in their own communities to those with lower levels of education, who might not participate in a university-sponsored intervention but who may participate in a more grassroots-level intervention led by the trained participants.

## Conclusion

This study, which displayed strong evidence of high statistical reliability of the ARTQ, is the first test of reliability of this instrument in a predominantly AA sample with large numbers of AAs. Our analysis showed that the ARTQ exhibited strong evidence of high statistical reliability when used in a study comprising AAs. This could be replicated to confirm the reliability of the ARTQ in other minority populations thereby laying the foundation for its use in these diverse populations. In future studies, the ARTQ could be used to assess the perceptions of other populations towards randomized trials. Such populations include but are not limited to those with lower education levels, low income levels, Latino or Hispanic communities and subgroups of AAs (different ancestral background). This instrument could also be useful in predominantly bi-lingual or non-English speaking populations within the US who have been shown to have low clinical trial participation rates (Fisher and Kalbaugh 2001).

It could be administered to potential trial participants to identify those whose trial perceptions indicate that they could benefit from participating in a clinical trials education program. Those who score low on the ARTQ may benefit the most from a cancer clinical trial education intervention to enhance their perceptions of trials and to potentially increase rates of clinical trial enrollment. Alternatively, the education program and the ARTQ could be incorporated into standard trial recruitment procedures.

The ARTQ results could thus foster communication about trials and highlight areas where investigators could spend additional time describing the trial design and responding to questions about specific aspects or components of the trial. Some of these aspects include information on any previous studies, possible risks, standard of care, availability of insurance coverage, and availability of holistic health care.
